# Liquidus Diagram of the *Ba-Y-Cu-O* System in the Vicinity of the *Ba_2_YCu_3_O_6+x_* Phase Field

**DOI:** 10.6028/jres.103.023

**Published:** 1998-08-01

**Authors:** Winnie Wong-Ng, Lawrence P. Cook

**Affiliations:** National Institute of Standards and Technology, Gaithersburg, MD 20899-0001

**Keywords:** Ba_2_YCu_3_O_6+_*_x_*, liquid compositions, liquid immiscibility, phase equilibria, primary phase field, superconductors

## Abstract

This paper describes the melting equilibria in the vicinity of the high *T*_c_ phase Ba_2_YCu_3_O_6+_*_x_*, including evidence for two Ba-Y-Cu-O immiscible liquids. Melting equilibria have been investigated in purified air using a combination of differential thermal analysis (DTA), thermogravimetric analysis (TGA), powder x-ray diffraction (XRD), MgO wick entrapment of liquid for analysis, scanning electron microscopy (SEM) coupled with energy dispersive x-ray analysis (EDS), and hydrogen reduction for determination of copper oxidation state. For relatively barium-rich compositions, it was necessary to prepare the starting materials under controlled atmosphere conditions using BaO. A liquidus diagram was derived from quantitative data for the melts involved in various melting reactions. In general the 1/2(Y_2_O_3_) contents of the melts participating in these equilibria were low (mole fraction <4 %). The primary phase field of Ba_2_YCu_3_O_6+_*_x_* occurs at a mole fraction of <2.0 % 1/2Y_2_O_3_ and lies very close along the BaO-CuO*_x_* edge, extending from a mole fraction of ≈43 % CuO to a mole fraction of ≈76 % CuO. It is divided by a liquid miscibility gap and extends on either side about this gap. The topological sequence of melting reactions associated with the liquidus is presented as a function of temperature. Implications for the growth of Ba_2_YCu_3_O_6+_*_x_* crystals are discussed.

## 1. Introduction

Extensive international research efforts since 1986 have led to a large body of information concerning the crystal chemistry and phase equilibria of the Ba-Y-Cu-O system. This is particularly true for the subsolidus relationships [1–4], as they are essential for the preparation of the high *T*_c_ compound Ba_2_YCu_3_O_6+_*_x_* in single-phase, crystalline form. The liquidus, although important for crystal growth and melt processing, remains controversial. Various authors have discussed the primary phase field for Ba_2_YCu_3_O_6+_*_x_*, and have investigated univariant reactions in the phase diagram near the CuO-rich corner, including the characterization of reaction products using x-ray diffraction, scanning electron microscopy (SEM), and electron probe microanalysis [5–22]. Numerous studies related to the melting and solidification behavior of the Ba_2_YCu_3_O_6+_*_x_* phase have also been conducted [23–30]. Melt processing investigations of this high *T*_c_ material, with important commercial applications, constitute a major activity within the high *T*_c_ superconductor research community [31–38].

Much of the relevant previous work concerning melting equilibria of the Ba-Y-Cu-O system can be summarized as follows. Roth et al.[1,2] studied the subsolidus phase relations extensively and provided a preliminary estimate of the liquidus. Aselege and Keefer [5] were the first to report a liquidus based on a reaction melting sequence; subsequently, Lay and Renlund [6] explored the effect of oxygen partial pressure. Ullman and McCallum [7] extrapolated the liquidus to the lanthanide-substituted systems at various oxygen partial pressures. Nevriva et al. [8] used a soaking technique to separate melt from solid and analyzed the residue by a difference method in order to obtain an approximate liquidus diagram. Rian [20] calculated a liquidus diagram using a thermochemical approach. Scheel and Licci [21] reviewed the literature data and constructed a partial liquidus for purposes of crystal growth. Osmura and Zhang [12] proposed a detailed reaction sequence which differs from others wherein the Ba_2_YCu_3_O_6+_*_x_* phase melts to form two solids plus a liquid. Krabbes et al. [17–19] applied the soaking technique to obtain data for a semi-quantitative liquidus and reaction sequence. Wong-Ng and Cook [39, 40] developed an MgO wicking method and used quantitative energy dispersive x-ray spectrometry (EDS) to determine melt compositions and reaction sequences associated with the liquidus. They also reported a possible immiscible liquid field in the Ba-Y-Cu-O system [13–16].

As discussed in Ref. [21], the sizes and shapes initially reported for the primary phase field of the high *T*_c_ Ba_2_YCu_3_O_6+_*_x_* phase differed substantially from one another. Such variations among reported liquidii can be generally attributed to the limited melting data they were based on, a situation which arises mainly because of the experimental complexity of this system. Difficulties include atmospheric contamination, the tendency of liquids to creep out of experimental containers, corrosion of containers, nonquenchability of the liquid phase, and the complexity of the phase assemblages. Another difficulty has been the lack of adequate software for performing quantitative EDS analysis in situations such as this where complex spectral manipulations are required to achieve optimum results. A comprehensive experimental procedure, including the use of special materials handling methods, has been developed in our laboratories to overcome some of the difficulties mentioned above [39, 40]. In addition, the availability of a quantitative analysis program developed by Fiori et al. [41], which incorporates flexibility based on a comprehensive treatment of the x-ray physics [42] has made it possible to obtain the necessary quantitative data on melt compositions. Recently, this overall procedure has been successfully applied in our laboratory to characterize the events associated with eutectic melting in the Ba-Y-Cu-O system [43]. This work, together with other published studies [24, 25], has indicated a quantitatively low ytrrium content for liquids in equilibrium with Ba_2_YCu_3_O_6+_*_x_*.

**Fig. 1:**
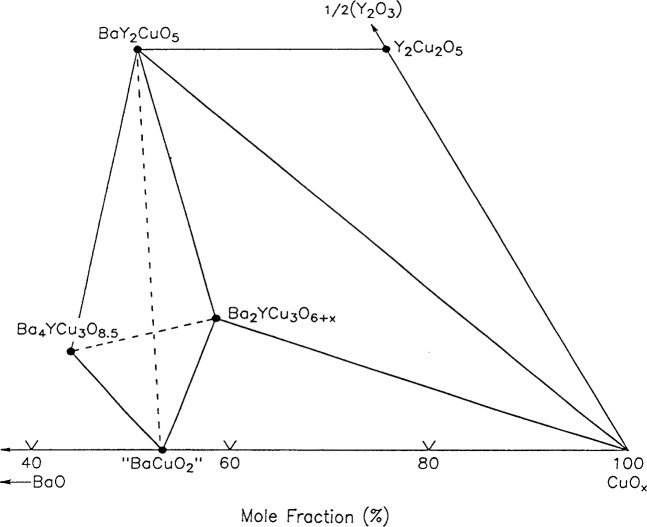
BaO-1/2Y_2_O_3_-CuO subsolidus phase relationships in air. When the starting samples were prepared from BaO, the stable tie line was Ba_4_YCu_3_O*_x_*-Ba_2_YCu_3_O_6+_*_x_* instead of BaY_2_CuO_5_-BaCuO_2_.

The present investigation is a continuation of our effort to understand the melting equilibria of the Ba-Y-Cu-O system in purified (CO_2_- and H_2_O-scrubbed) air, with emphasis on the region near the high *T*_c_ Ba_2_YCu_3_O_6+_*_x_* phase. The goal of this paper is to report our current best estimate of the primary phase field of the high *T*_c_ phase Ba_2_YCu_3_O_6+_*_x_*, and its boundaries with the neighboring phase fields of BaY_2_CuO_5_, Y_2_O_3_, Y_2_Cu_2_O_5_, CuO, Ba_4_YCu_3_O_8.5_ and BaCuO_2_. Figure 1 shows a partial subsolidus diagram which has served as the basis for our liquidus studies. Two alternative tie line connections for the four phases Ba_4_YCu_3_O_8.5_, Ba_2_YCu_3_O_6+_*_x_*, BaY_2_CuO_5_, and BaCuO_2_ are shown. When the starting materials used were oxides, rather than carbonates, and the sample preparation and annealing process avoided the presence of CO_2_, the pervoskite phase present in the Ba-rich region was Ba_4_YCu_3_O_8.5_ [9]. Under these conditions the tie-line connection was found to be between Ba_4_YCu_3_O_8.5_ and Ba_2_YCu_3_O_6+_*_x_*, instead of between BaY_2_CuO_5_ and BaCuO_2_, as reported under other conditions [9, 12]).

## 2. Experimental Procedures

### 2.1 Sample Preparation

Samples for this investigation were prepared by using the solid state sintering technique. These compositions were chosen according to one of several desired locations: 1) along the join between two compounds; 2) within a three phase equilibrium region; 3) corresponding to a ternary compound; 4) corresponding to one or a mixture of postulated immiscible liquids. In general, a mixture of research grade BaCO_3_, Y_2_O_3_ and CuO powders was weighed, homogenized under acetone, pressed into a pellet, and calcined at 850 °C overnight to decompose most of the carbonate. Further higher temperature calcinings, including intermediate crushings and grindings, were then carried out, depending upon the location of the composition within the phase diagram. For example, to study the eutectic melting of the Ba-Y-Cu-O and Ba-Cu-O systems, the highest processing temperature was kept below 880 °C (approximately the reported eutectic temperature [1, 5]), in order to avoid possible premelting of the sample. For other samples such as single phase Ba_2_YCu_3_O_6+_*_x_*, and BaY_2_CuO_5_, after an initial heat treatment at 850 °C, further annealings were performed at 900 °C and 930 °C for about 4 days with intermediate grindings. Pre-equilibration of samples in purified air was performed before the quenching experiments. Powder x-ray diffraction was conducted to ensure the presence of the desired phase(s). For barium-rich compositions, starting materials were prepared using BaO, synthesized by vacuum calcining of the carbonate. Powder x-ray diffraction (XRD) showed the BaO to be single phase, and weight loss data indicated it to be at least 99.99 % carbonate free. For the BaO-containing samples, all operations, including weighing, calcining, differential thermal/thermogravimetric analysis (DTA/TGA), and XRD were completed under controlled atmosphere conditions in gloveboxes or other apparatus, as appropriate. Table 1 lists the compositions prepared which were pertinent to this study.

### 2.2 Apparatus and Methodology

The various steps in our experimental procedure [39] can be summarized as follows: (1) DTA/TGA studies to obtain an indication of thermal events and oxygen loss, (2) annealing in purified air followed by quenching of samples in liquid nitrogen-cooled helium, (3) XRD characterization of solid phases present, (4) scanning electron microscopy (SEM) characterization and x-ray mapping to determine the microstructures of the quenched materials, (5) equilibration with porous MgO wicks in order to capture the liquid formed, (6) quantitative SEM/energy dispersive spetrometry (EDS) of the composition of the quenched wick experiments, and (7) thermogravimetric hydrogen reduction to obtain the oxygen content. Quench experiments and DTA/TGA experiments were carried out in purified air, unless otherwise noted.

Simultaneous DTA/TGA was completed using high density MgO crucibles and an α-alumina or a powdered Pt reference. The system was calibrated against the melting point of Au (1063 °C). The DTA/TGA system was arranged to allow a fresh flow of purified air past the sample during analysis. DTA and DTG event onset temperatures were determined by the usual method which utilized the intersection of the base line with the extension of the linear region of the rising peak slope. Event temperatures were estimated to have standard uncertainties (1.0 estimated standard deviations) of less than 10 °C. For most experiments, a heating rate of 4 °C/min was used.

Temperatures of melting events have been selected based on the first heating cycle of carefully equilibrated and oxidized mixtures. Data from the first heating cycle was considered to be the closest indication of the true equilibrium temperature of the particular melting event because of the following observations. When samples containing BaCuO_2_ were subjected to DTA/TGA experiments involving multiple heating cycles, the melting events of the second and subsequent cycles were always observed at a lower temperature when compared to the first one. This was interpreted as a result of the presence of metastable BaCu_2_O_2_, which melts at a lower temperature than BaCuO_2_. It was nearly impossible to completely reoxidize BaCu_2_O_2_ once it had formed during the initial melting cycle, even though this phase is not stable in air in solid form. This appears to be associated with the kinetics of oxygen diffusion through the oxidized surface layer of crystallized melt products at subsolidus temperatures. It is likely that some cation reordering is also necessary for complete reoxidation to the original premelted state.

For quenching studies, the samples were contained in small MgO crucibles suspended from the thermocouple assembly by thin Pt wires. The details of the furnace are described by Wong-Ng and Cook [39]. After insertion of the sample, the furnace was valved off, and flushed with purified air. The sample was then equilibrated in the hot-zone of the furnace in the presence of flowing purified air. At the conclusion of the experiment, the gate valve at the bottom was quickly opened, and current was passed through the thin Pt suspension wires, causing them to melt. The sample dropped into the liquid nitrogen (LN_2_)-cooled copper cold well, through which LN_2_-chilled helium was flowing at a rapid rate.

After equilibrating and quenching of the samples, parts of each sample were crushed and subjected to powder x-ray diffraction. An automated diffractometer equipped with a theta-compensated slit and CuKα radiation (45 kV and 40 mA) was used. The radiation was detected by a scintillation counter and solid-state amplifier. Commercially available software and the reference x-ray diffraction patterns of the Powder Diffraction File [44] were used for performing phase identification. A sealed cell designed by Ritter [45] was used for x-ray analysis of the air- and moisture-sensitive samples. This cell was filled with sample within a glove-box, then transferred to the diffractometer. Reaction with atmospheric CO_2_ and H_2_O during analysis was prevented by the “O”-ring seal of the cell. The design of the cell minimized signal absorption by employing an x-ray transparent window.

To capture liquid free of any primary crystalline phases for SEM/EDS analysis, a sintered MgO wick with open porosity was placed in the MgO crucible with the appropriate sample. During the heat treatment, which took place at a temperature a few degrees above the particular melting event of interest, liquid was drawn into the wick by capillary action. Due to the filtering action of the small (<5 μm to 10 μm) openings in the wick, primary solid phases could not enter the capillary. During the quench, this liquid was retained in the wick, thus making a representative sample of the melt available for analysis. This approach works only for sampling melts of events where the observed liquid is the first liquid to appear for the given bulk composition. Otherwise, a range of liquid compositions is likely to be generated.

To analyse quenched liquids, smooth surfaces of samples were first prepared. Special attention was paid to the SEM operating parameters (15 kV, 41° optimum takeoff angle in our instrument) to miminize absorption of the YLα x rays and their interaction with the MgO matrix. Data were collected using the broad beam method described previously [39, 40] and reduced using the usual atomic number/absorption/fluorescence (ZAF) correction procedure [42] via the DTSA quantitative data reduction program [41]. This program allows highly accurate background correction and peak stripping by the use of an advanced digital filtering algorithm. The capabilities of this program were essential for analysis of Y at low levels in the infiltrated MgO wicks. The standards used were Ba_2_YCu_3_O_6+_*_x_* and BaCuO_2_. Uncertainties are estimated as follows: for mole fractions >0.1, <0.005 expanded uncertainty (coverage factor *k* = 2 and thus a two standard deviation estimate); for mole fractions <0.1, <0.01 expanded uncertainty.

Quantitative TGA was used to study the oxidation/reduction associated with the eutectic melting reaction and the melting of BaY_2_CuO_5_ by using the hydrogen reduction method. The samples were heated gradually in the TGA instrument in a flowing mixture of Ar of volume fraction of 95 % and H_2_ of 5 %, and the point at which the weight loss leveled off was noted. The total weight loss gave the oxygen content of the CuO*_x_* in the sample. X-ray analysis of products generated by this method has generally confirmed reduction to metallic Cu plus BaO, Y_2_O_3_, and other intermediate binary phases. The Cu^+1^/(Cu^+1^ + Cu^+2^) ratio in the melt was determined by simply noting the weight loss as the sample went through complete melting, and relating this back to the ratio determined for the starting material.

As noted above, we have chosen MgO to be the material in contact with the Ba-Y-Cu-O samples. The selection of MgO containers and MgO wicks was based on the relatively high resistance of this material to attack by alkaline earth cuprates. We have not found another practical material of comparable resistance to a large range of cuprate compositions. While MgO in contact with cuprate liquids for extended periods of time has shown a slight indication of copper diffusion, this took place only within a thin skin directly in contact with the melt. The diffusion of CuO into the MgO has always been over distances of less than 1 μm. A relatively minor interaction of this type with the high density MgO crucibles used in these experiments did not have a quantitatively significant effect on the sample bulk compositions. Any tendency for CuO diffusion into the MgO crucibles was minimized by the relatively short durations of the quench experiments (typically 1.0 h at a particular temperature). In the majority of samples, the chemical potential of CuO was considerably lowered relative to pure CuO, thus further reducing the tendency for interaction between the MgO crucibles and the CuO component of the samples.

For the porous MgO wick experiments, since a broad beam analysis technique was used, any local diffusion of copper at the surface of the MgO grains in the interior of the wicks did not affect the determination of the overall copper content of the liquid phase sampled by the wick. After absorption in the wick by capillary action, the copper content of the sample (wick plus absorbed liquid) remained constant, in spite of any minor redistribution at the grain boundaries. The outermost limits of the wicks were not included in the analyses. The overall size of the wicks relative to the amount of sample was such that any diffusion of copper at the surface of the wicks did not significantly affect the sample bulk composition. The penetration of liquid into the wicks by capillary action occurred on a much more rapid time scale than any diffusion of copper into the MgO, and therefore the wick sampling method was not affected by CuO diffusion. In summary, regarding our use of MgO, we have found it possible to minimize any interaction with the Ba-Y-Cu-O samples, so that it has served as a very practical and useful material for containers and wicks for melt sampling.

## 3. Results and Discussion

### 3.1 Derivation and General Features of the Liquidus Diagram

Table 1 gives the DTA initial melting temperatures of the compositions investigated. Annealing and quench temperatures, crystalline phases (from XRD) and melt compositions (from EDS) are shown in Table 2 for selected experiments. The interpretation of crystalline solids as primary phases vs crystallized melts was based on comparing the XRD results before and after the melting events, and in some cases, by x-ray mapping and microstructural analysis of quenched samples.

**Fig. 2:**
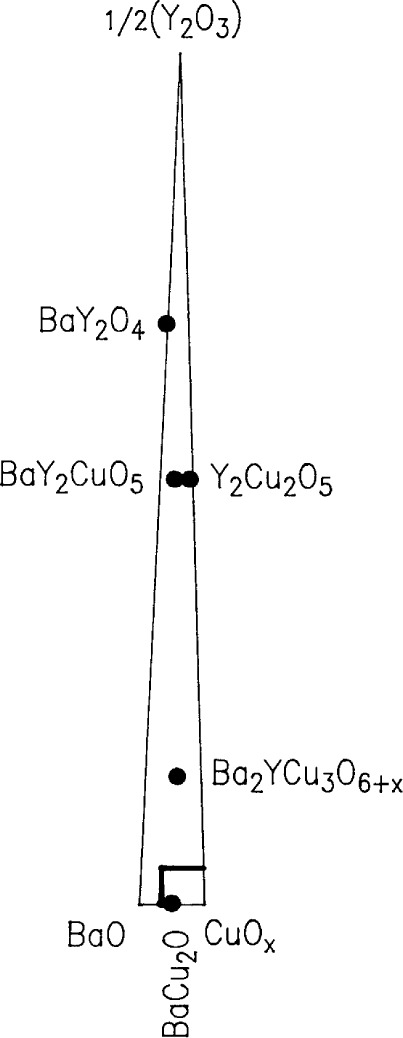
Composition triangle used as the basis for Figs. 3 and 7, showing “stretched” 1/2(Y_2_O_3_) coordinates. The small box indicates region of interest.

**Fig. 3:**
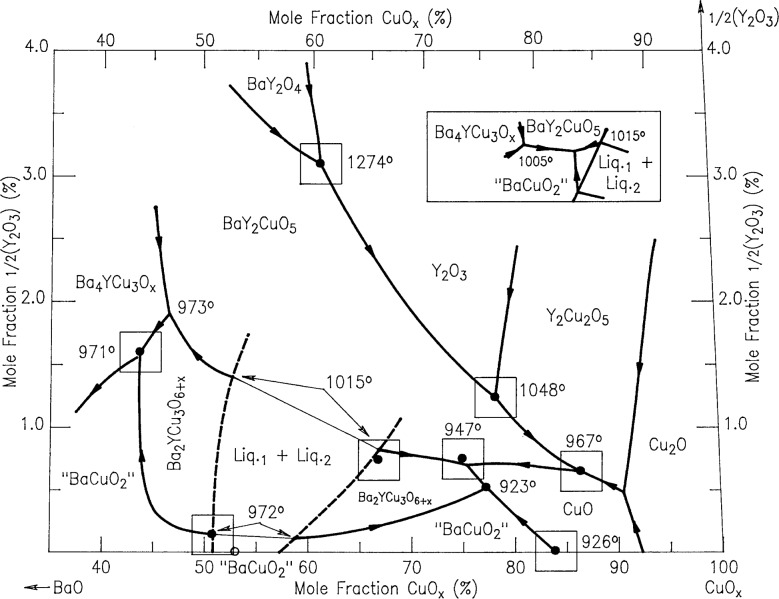
The Ba-Y-Cu-O liquidus showing the primary phase fields adjacent to the Ba_2_YCu_3_O_6+_*_x_* primary field, as defined by invariant (in air) melt compositions. The inset shows schematically the phase relations at the BaO-rich termination of the Ba_2_YCu_3_O_6+_*_x_* field when BaCO_3_, rather than BaO, was used to prepare the starting materials.

In order to plot our liquidus data for the Ba-Y-Cu-O system, it was necessary to use a coordinate system which magnified the yttrium oxide contents of the liquids, all of which were below a mole fraction of 4 %. This was accomplished by “stretching” the customary ternary composition triangle, as indicated in Figure 2, where the small compositional area of interest is shown relative to the positions of the relevant solid phases. Using this coordinate system, Fig. 3 shows the liquidus diagram of the Ba-Y-Cu-O system in the vicinity of the Ba_2_YCu_3_O_6+_*_x_* phase field, as based on data summarized in Table 2. The phase fields of BaY_2_O_4_, Y_2_O_3_, BaY_2_CuO_5_, Ba_4_YCu_3_O*_x_*, Y_2_Cu_2_O_5_, Cu_2_O, nominal BaCuO_2_ (“BaCuO_2_”), and CuO are also shown. While the horizontal axis in Fig. 3 extends from a mole fraction of 35 % CuO to a mole fraction of 100 % CuO, the vertical axis extends from a mole fraction of 0 % yttria (as 1/2Y_2_O_3_) to a mole fraction of 4 % yttria (as 1/2Y_2_O_3_). The yttria apex of this diagram would therefore be at a position 25 times as high as the diagram shown. The Ba_2_YCu_3_O_6+_*_x_* superconductor, which contains a mole fraction of 16.7 % 1/2Y_2_O_3_, would plot off the top of the diagram at a height of more than four times that of the diagram. The filled dots in Fig. 3 indicate quantitative EDS analyses of the melt phases participating in the melting equilibria. In a general sense, these equilibria are univariant. However, at constant oxygen pressure (*p*(O_2_) = 0.21×10^5^ Pa), they are invariant, i.e., they plot as points on the phase diagram. The boxes around the dots illustrate the standard uncertainty (i.e., one standard deviation) associated with the melt analyses. Data for the 923 °C eutectic event were taken from Ref. [43].

The most significant departure from previously reported liquidus diagrams [5–22] is the two liquid field of immiscibility shown on the left hand side of the diagram. However, before a detailed discussion of the features of this diagram is possible, a brief description of the melting of the phases BaY_2_CuO_5_, BaCuO_2_ and Ba_2_YCu_3_O_6+_*_x_*, and a presentation of the evidence for liquid immiscibility are necessary. Our results demonstrated that melting in these regions may be more complicated than previously thought.

### 3.2 Melting of the “Green Phase” BaY_2_CuO_5_

Our observations on the peritectic melting reaction of BaY_2_CuO_5_ differed from literature results [1, 5, 6, 17]. Instead of the reported melting to one solid (Y_2_O_3_) and liquid, we observed two solids plus liquid as products.

**Fig. 4:**
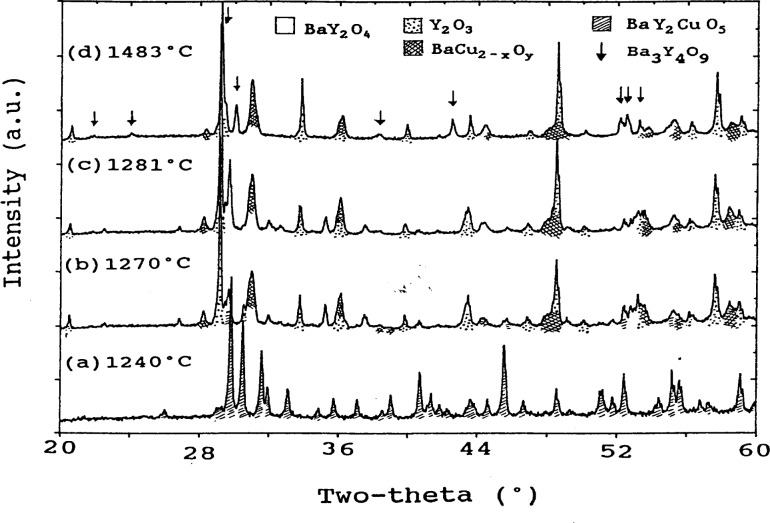
X-ray diffraction patterns of BaY_2_CuO_5_ quenched from 4 different temperatures.

In order to characterize the melting behaviour of BaY_2_CuO_5_, several experiments were performed. Figure 4 shows the x-ray diffraction patterns of samples quenched at 1240 °C, 1270 °C, 1281 °C, and 1483 °C. The two higher temperature experiments indicated the formation of BaY_2_O_4_ and Y_2_O_3_. This is interpreted to mean that melting takes place according to the following reaction:

$${\text{BaY}}_{2}{\text{CuO}}_{5}\to {\text{BaY}}_{2}O_{4}+Y_{2}O_{3}+L+O_{2}.$$

A small amount of Ba_2_YCu_3_O_6+_*_x_* was also observed, which originated, along with barium cuprates, from crystallization of the melt phase. Above 1483 °C the BaY_2_O_4_ phase decomposed to form Ba_3_Y_4_O_9_, as shown in Fig. 4d.

**Fig. 5:**
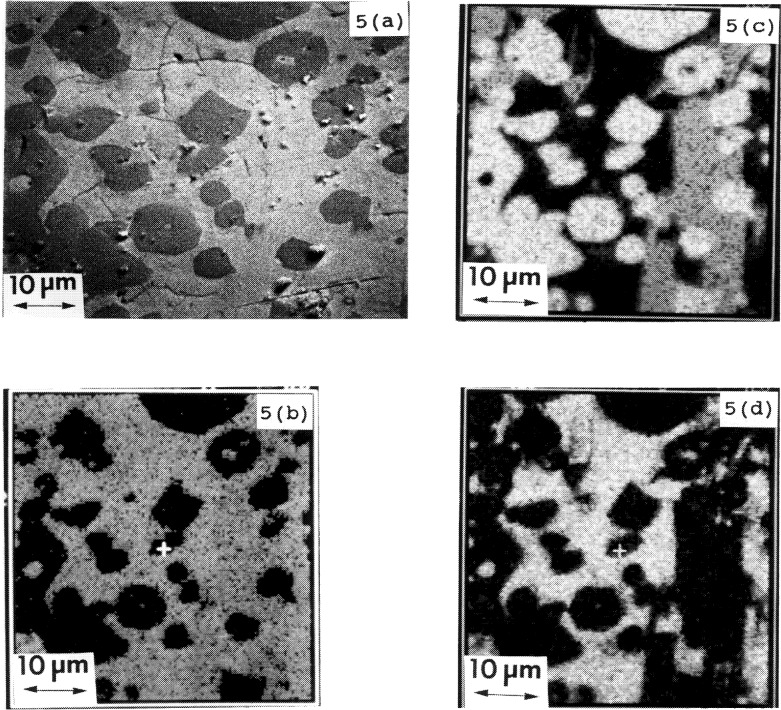
Microstructure of the “green phase” (BaY_2_CuO_5_) melt. (a) Secondary electron image showing Y_2_O_3_ primary crystals, and x-ray maps of (b) BaLa, (c) YLa, and (d) CuKα at 20 kV.

To confirm that BaY_2_O_4_ was a primary phase instead of a quench product, x-ray mapping studies of a sample quenched from just above the melting point were completed. An SEM micrograph of a polished section of the residual from the 1274 °C melting experiment is shown in Fig. 5a. At 1274 °C, the primary Y_2_O_3_ grains and the interstitial liquid were obvious. The melt was found to concentrate in the intergranular area. Due to the similarity of average atomic number of BaY_2_O_4_, Ba_2_YCu_3_O_6+_*_x_*, BaCuO_2_ and BaY_2_CuO_5_, it was not easy to differentiate the presence of BaY_2_O_4_ in the micrograph. For this reason x-ray mapping was used for confirmation. X-ray maps of the distribution of Ba, Cu, and Y in the area corresponding to the SEM micrograph (Fig. 5a) are shown in Figs. 5b to 5d. The melt was expected to be of high Cu concentration relative to the primary solid phases, and the CuKα map indeed shows an intersititial melt surrounding the primary Y_2_O_3_ phase. The YLa map shows the presence of three phases: the liquid phase contained only very little Y, as was also indicated by EDS analysis of the associated wick; the grains with high Y signal indicate the Y_2_O_3_ phase; and the large “grey” grain which has a well defined morphology indicates the presence of primary BaY_2_O_4_. In the BaLamap, the slightly shadowed region indicating a phase with relatively lesser Ba corresponds to the same BaY_2_O_4_ grain. The intergranular melt phase is enriched in Ba and Cu. In summary, large crystals of Y_2_O_3_ and BaY_2_O_4_, which can be distinguished microstructurally from the interstitial melt, have been observed. Therefore, we suggest that BaY_2_CuO_5_ melts to two primary crystals instead of one. This result is consistent with the melt composition, which lies off to the CuO-rich side of the extension of the Y_2_O_3_ -BaY_2_CuO_5_ tie-line (join).

### 3.3 Composition and Melting of BaCuO_2_

At temperatures below 900 °C, at the BaCuO_2_ composition homogeneous material can be prepared, making it of use as an EDS standard. However at higher temperatures, i.e., at temperatures above about 900 °C in air, we have concluded that it is non-stoichiometric. Briefly, this is based on the following: (1) EDS analysis of well-formed cubes of BaCuO_2_ grown from melt by slow-cooling at ≈1 °C/min of a nominal BaCuO_2_ composition in a MgO crucible indicated a composition of Ba_0.94_Cu_1.06_O*_x_*; (2) the structure determination of Paulus et al. [46] indicated a disordered structure with a formula corresponding to Ba_0.93_Cu_1.07_O*_x_*, and (3) x-ray diffraction analysis of extensively annealed compositions nominally on the Ba_2_YCu_3_O_6+_*_x_* – BaCuO_2_ tie line always indicated the presence of a third phase.

The identity of the third phase produced in nominal Ba_2_YCu_3_O_6+_*_x_* - BaCuO_2_ compositions was found to depend on whether the mixture was prepared using BaO, or via the usual method of calcining carbonate mixtures repeatedly in air, followed by extensive equilibration in purified air. For the BaO route, the third phase was Ba_4_YCu_3_O*_x_*, whereas for the carbonate/calcining route, the third phase was BaY_2_CuO_5_. Notwithstanding the importance of third phase identity for the tie line distribution, as discussed below, the implications for BaCuO_2_ nonstoichiometry are similar regardless of whether Ba_4_YCu_3_O*_x_* or BaY_2_CuO_5_ was the additional phase. Given that Ba_2_YCu_3_O_6+_*_x_* is known to be stoichiometric in cation content to within analytical limits, the presence of the third phase in tie line mixtures requires BaCuO_2_ to be nonstoichiometric in the direction of excess CuO.

It is generally known that BaCuO_2_ has a cubic *Im3m* structure with a large cell of edge length *a* ≈1.82 nm. The structure is reported to be ordered in Ba and Cu but non-stoichiometric in oxygen, and the structure is complicated by the presence of large Cu-O polyhedral clusters [47–50]. Paulus et al.[46] reported a partially disordered version of the structure of this compound and the chemical formula was determined to be Ba_0.93_Cu_1.07_O*_x_*. We believe that this composition is consistent with our experimental results.

To investigate melting, the “BaCuO_2_” composition (Ba_0.94_Cu_1.06_O*_x_*) was prepared using BaO, and after several heat treatments at 900 °C, the x-ray pattern indicated single phase material. The compound was found to melt at 984 °C in air, possibly to two liquids, as discussed below.

It is likely that the phase region near the BaCuO_2_ composition is complicated by the presence of a solid solution or by polymorphism, especially at lower temperatures. However, for simplicity this phase has been represented in Fig. 3 and the isothermal sections discussed later as the point compound “BaCuO_2_” at a composition with a mole fraction of 47 % BaO, and a mole fraction of 53 % CuO. The effect of this simplification on the phase diagram topologies, particularly with respect to the stability of the Ba_2_YCu_3_O_6+_*_x_* phase, is judged to be small. Non-stoichiometry and structure of this “BaCuO_2_” phase have been discussed in more detail elsewhere [51].

### 3.4 Melting of Ba_2_YCu_3_O_6+_*_x_*

Melting of the Ba_2_YCu_3_O_6+_*_x_* phase has been studied extensively. To date, at least three alternative melting schemes have been proposed:

$$\begin{array}{ll} \left(i\right) & {\text{Ba}}_{2}{\text{YCu}}_{3}O_{6+x}\to {\text{BaY}}_{2}{\text{CuO}}_{5}+L\\ \left(\mathrm{ii}\right) & {\text{Ba}}_{2}{\text{YCu}}_{3}O_{6+x}\to {\text{BaY}}_{2}{\text{CuO}}_{5}+{\text{Ba}}_{4}{\text{YCu}}_{3}O_{x}+L\\ \left(\mathrm{iii}\right) & \begin{array}{l} \text{Stage}\;1.\;{\text{Ba}}_{2}{\text{YCu}}_{3}O_{6+x}\to {\text{BaCuO}}_{2}+L\\ \text{Stage}\;2.\;{\text{BaCuO}}_{2}+L\to {\text{BaY}}_{2}{\text{CuO}}_{5}+L \end{array} \end{array}$$

Reaction (i) corresponds to the widespread view that Ba_2_YCu_3_O_6+_*_x_* melts to BaY_2_CuO_5_ and liquid. As an alternative (ii), Osamura and Zhang [12] suggested this phase melts incongruently to two solids (pervoskite phase Ba_4_YCu_3_O*_x_* and the “green phase,” BaY_2_CuO_5_) plus liquid. According to alternative (iii), Rodriguez et al. [32, 33] suggested on the basis of their high temperature x-ray study a two stage mechanism in which Ba_2_YCu_3_O_6+_*_x_* first melts to BaCuO_2_ plus a liquid with a high Y content, then BaCuO_2_ reacts with this liquid to form BaY_2_CuO_5_.

There have been various reports concerning the observation of a small thermal event taking place at about 950 °C during the DTA experiments on Ba_2_YCu_3_O_6+_*_x_* [6, 7, 43]. These have been attributed to the reaction of Ba_2_YCu_3_O_6+_*_x_* with second phases at the grain boundaries, such as BaCuO_2_ and CuO. Our DTA experiments on a Ba_2_YCu_3_O_6+_*_x_* sample prepared from BaO and handled in CO_2_- and H_2_O-scrubbed air showed the absence of this peak. Therefore it is most likely that the occurrence of this peak was associated with the presence of a carbonate-containing phase. Further support for this comes from the observations of Aselage [26], who concluded that an endothermic event observed in samples of Ba_2_YCu_3_O_6+_*_x_* near 940 °C in air was due to the presence of BaCO_3_, CuO, and other impurities.

**Fig. 6:**
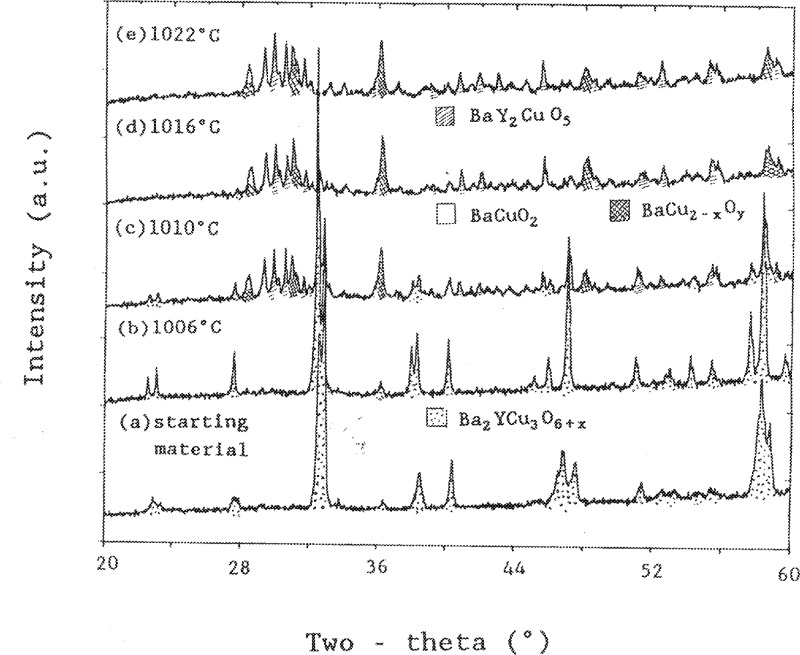
X-ray diffraction patterns from a sequence of quench experiments illustrating melting of Ba_2_YCu_3_O_6+_*_x_*.

X-ray patterns of Ba_2_YCu_3_O_6+_*_x_* samples quenched from temperatures of 1006 °C to 1022 °C are shown in Fig. 6. It is evident that only Ba_2_YCu_3_O_6+_*_x_* was present until above 1006 °C. Above this temperature, BaY_2_CuO_5_ appeared along with barium cuprate phases. The BaY_2_CuO_5_ originated as a solid decomposition product of Ba_2_YCu_3_O_6+_*_x_*, and the barium cuprates originated from recrystallized melt. This interpretation is based on the combination of powder x-ray diffraction studies of the residuals left behind as the Ba_2_YCu_3_O_6+_*_x_* melted, and comparison with liquid-infiltrated MgO wicks from the same experiments. Our analysis of the wicks showed that the Y content of the liquid was always low throughout the range over which we have studied the melting of Ba_2_YCu_3_O_6+_*_x_*. Quantitative analyses of liquids produced by Ba_2_YCu_3_O_6+_*_x_* just above its initial melting (which were trapped by porous MgO wicks) gave melt compositions plotting off the line connecting the green phase and Ba_2_YCu_3_O_6+_*_x_*, with an approximate CuO*_x_*:BaO: 1/2Y_2_O_3_ amount of substance ratio of 66.0:33.3:0.7. The same conclusion has been reached from several experiments on the initial melting of Ba_2_YCu_3_O_6+_*_x_*. When a much higher temperature (1030 °C to 1050 °C) was used, the melting produced liquids much closer to the extended BaY_2_CuO_5_ - Ba_2_YCu_3_O_6+_*_x_* join; however, these were not representative of the first liquid composition to appear on melting of Ba_2_YCu_3_O_6+_*_x_*. Given this initial liquid composition, mass balance considerations dictate that the melting of Ba_2_YCu_3_O_6+_*_x_* cannot be simply to a mixture of BaY_2_CuO_5_ and liquid. The implication of the mass balance analysis is that there must be a third phase, X, in order to balance the melting reaction,

$${\text{Ba}}_{2}{\text{YCu}}_{3}O_{6+x}\to {\text{BaY}}_{2}{\text{CuO}}_{5}+L+X+O_{2}.$$

Phase X must have a composition which lies to the left (barium-rich side) of the extended Ba_2_YCu_3_O_6+_*_x_*+ BaY_2_CuO_5_ join. There are two possibilities for its identity—either it is a solid, such as a barium cuprate, or Ba_4_YCu_3_O*_x_*, or it is another liquid phase. The latter possiblity requires the existence of a liquid immiscibility field. If phase X is a solid barium cuprate phase, possible candidates are Ba_2_Cu_3_O_5_, Ba_3_Cu_5_O_8_ [50] and “BaCuO_2_.” The Ba_3_Cu_5_O_8_ phase has not been proved to be a stable compound and may actually have been the Ba_2_Cu_3_O_5_ phase instead [52]. Since Ba_2_Cu_3_O_5_ decomposes to BaCuO_2_ above 800 °C [51], it is not a candidate product during the melting of Ba_2_YCu_3_O_6+_*_x_*. If X is “BaCuO_2_,” then the tie line between “BaCuO_2_” and Ba_2_YCu_3_O_6+_*_x_* should remain stable up to the melting point of Ba_2_YCu_3_O_6+_*_x_*. This was not found to be the case when carbonate-derived starting materials were used, as this tie line was broken to yield BaY_2_CuO_5_ + liquid at a temperature between 1000 °C and the melting of Ba_2_YCu_3_O_6+_*_x_*. With starting materials derived from BaO, a similar conclusion was reached regarding the breaking of the tie line, although the products were different; in this case corresponding to Ba_4_YCu_3_O*_x_* + liquid. If phase X is Ba_4_YCu_3_O*_x_*, it should be present either in x-ray diffraction patterns or in x-ray compositional mapping; neither type of evidence was found in melted Ba_2_YCu_3_O_6+_*_x_* samples, despite a thorough search. The remaining possibility is the presence of a second liquid, and consequently a fourth Ba_2_YCu_3_O_6+_*_x_* melting alternative is proposed, as follows. At 0.1 MPa total pressure in CO_2_- and H_2_O-scrubbed air:

$$\begin{array}{cc} \left(\mathrm{iv}\right) & {\text{Ba}}_{2}{\text{YCu}}_{3}O_{6+x}\to {\text{BaY}}_{2}{\text{CuO}}_{5}+L_{1}+L_{2}+O_{2}. \end{array}$$

### 3.5 Evidence of Two Liquids

**Fig. 7:**
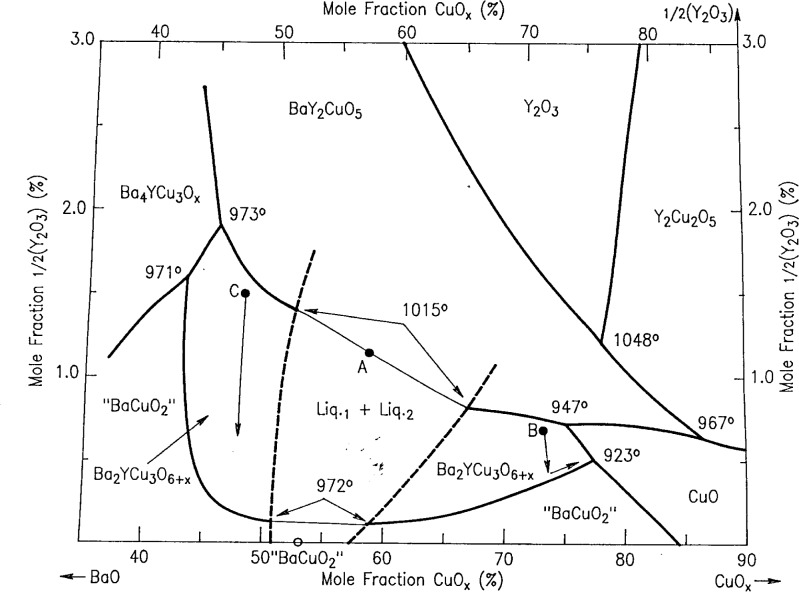
Enlarged portion of the liquidus of Fig. 3 showing the location of compositions A, B, and C, as described in the text. The approximate crystallization paths for compositions B and C are shown by the arrows.

**Fig. 8:**
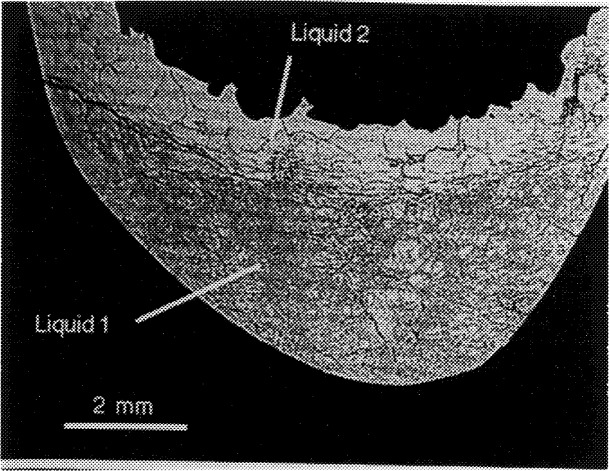
Liquid stratification in composition A, after heating at 1050 °C for one hour, as shown by a backscattered electron micrograph in the atomic number contrast mode.

Investigating the presence of two liquids in the Ba-Y-Cu-O system was a complicated process. Many experimental difficulties were encountered and among them, the incomplete removal of carbonate from the starting materials, even after extended annealing in purified air, was found to be a constant problem associated with the BaCuO_2_ phase. For most experiments, BaO was therefore used as a starting material in this region. As a direct test of the two-liquid field, two experiments in particular were important. In the first, a composition (amount of substance ratio Ba:Y:Cu = 40.85: 1.15: 58.00) lying in the center of the postulated two liquid field was prepared, as indicated by “A” in Fig. 7. This composition was carefully homogenized in a glovebox, equilibrated in purified air at 850 °C for 1 h, loaded into a 15 mm dia. MgO crucible, taken up to 1050 °C in purified air for 1 h (to insure complete melting at a temperature above the known melting points of Ba_2_YCu_3_O_6+_*_x_* and BaCuO_2_), and then furnace cooled. The resulting microstructure indicated complete melting. A cross section through the sample is shown in Fig. 8. Two layers are evident in this backscattered electron micrograph, taken in the atomic number contrast mode. The contrast results from the fact that the top layer is barium-rich relative to the bottom; there is no major difference in Y content. The higher average atomic number of the top layer suggests higher density at the top. This is counter to any expected gravity-driven stratification and suggests a surface tension-related effect, consistent with a two-liquid field. No evidence of crystallization at the temperature of the experiment or crystal settling was found. Estimated compositions of the separated layers lie within the boundaries of the miscibility gap of Fig. 3. This is consistent with a narrowing of the gap which is to be expected at temperatures above its base. Evidence of stratification was lacking in analogous melting/annealing experiments for compositions B and C, prepared on either side of the miscibility gap (see Fig. 7).

A second relevant experiment involved the preparation of a composition using BaO situated on the tie line between Ba_2_YCu_3_O_6+_*_x_* and Ba_0.94_Cu_1.06_O*_x_*. This composition is important because the tie line must be broken in order for an L_1_ + L_2_ coexistence. Our results showed that this tie line was indeed broken at 972 °C, producing a liquid on the BaO rich side of the tie line. This requires a compositionally balancing phase on the other side of the tie line, i.e., another more CuO-rich liquid.

An additional relevant observation is the existence of inhomogeneous melts over the range from a mole fraction of 50 % CuO to a mole fraction of 65 % CuO. This was apparent in wick samples of liquid from this region of the phase diagram, in contrast to other regions where different wick analyses from the same reaction specimen clustered closely together. Furthermore, invariant reaction temperatures near the left side of the diagram, involving the Ba_4_YCu_3_O*_x_* phase, occurred at a lower temperature than the melting of the Ba_2_YCu_3_O_6+_*_x_*. Because the invariant temperatures on the right side of the diagram are also lower than the melting of the Ba_2_YCu_3_O_6+_*_x_*, this requires a thermal maxiumum in the Ba_2_YCu_3_O_6+_*_x_* liquidus. Such a maximum could result from a stable solidus tie line originating at Ba_2_YCu_3_O_6+_*_x_* and connecting to another phase; however, as noted above, evidence for this was not found. The remaining alternative appears to be the presence of a two liquid field. Zhang et al. [53] in their binary diagram of the BaO-CuO system have indicated a narrow two liquid field near the BaCuO_2_ composition. Since our proposed immiscibility field in the Ba-Y-Cu-O system involves melts of low yttrium content, our two liquid field can be extended down to the binary join in a manner largely consistent with this literature data.

The fact that liquid immiscibility could be present in the ternary system, and yet not be observed by a large number of investigators can best be explained by the close similarity in composition of the two liquids. Since the miscibility gap is relatively narrow, it is likely that some of the properties, including color, density, and surface tension, are rather similar. There is relatively little driving force for segregation of the two liquids. Consequently, most sampling methods, especially for melts which have not been subject to long-term static heating, would tend to capture an intimate mixture of both liquids. This may explain why literature melt analyses [24, 25] give a composition for the melting of Ba_2_YCu_3_O_6+_*_x_* which plots on the extension of the line that joins Ba_2_YCu_3_O_6+_*_x_* and BaY_2_CuO_5_. Finally, it should be noted that observation of immiscible liquids is a well-known difficulty in several other oxide systems, especially the silicates, where abnormalities in the shape of the liquidus sometimes offer the best indication of a two-liquid field.

### 3.6 Details of the Liquidus Diagram

The left hand part of the diagram in Fig. 3 was constructed from data on mixtures made by using BaO. As shown in Fig. 3, the crystallization field of the Ba_2_YCu_3_O_6+_*_x_* phase occurs in two segments. Both segments of the field are entirely below the mole fraction level of 2.0 % 1/2Y_2_O_3_. The right hand segment of the Ba_2_YCu_3_O_6+_*_x_* field is bounded by the crystallization fields of BaY_2_CuO_5_, “BaCuO_2_,” and CuO. Under CO_2_- and H_2_O-scrubbed conditions with BaO-derived starting materials, the left hand segment was bounded by the Ba_4_YCu_3_O*_x_*, BaY_2_CuO_5_ and “BaCuO_2_” primary phase fields. When the carbonate/calcining route was employed, the Ba_4_YCu_3_O*_x_* phase field did not contact the Ba_2_YCu_3_O_6+_*_x_* phase field, as shown schematically in the inset to Fig. 3, because this was precluded by a BaY_2_CuO_5_ - “BaCuO_2_” tie line.

Figure 3 shows a total of two binary invariant equilibria and ten ternary invariant equilibria, as defined under conditions of *p*(O_2_) = 0.21×10^5^ Pa (air). The two binary invariant equilibria occur on the BaO-CuO*_x_* join, and correspond to the melting of “BaCuO_2_” at 984 °C to two liquids and to the “BaCuO_2_”/CuO eutectic at 926 °C. The ten ternary invariant equilibria involve the following phases: BaY_2_O_4_ / BaY_2_CuO_5_ / Y_2_O_3_ / Liquid (1274 °C), Ba_4_YCu_3_O*_x_* / BaY_2_CuO_5_ / Ba_2_YCu_3_O_6+_*_x_* / Liquid (972 °C), BaY_2_CuO_5_ / Ba_2_YCu_3_O_6+_*_x_* / Liquid 1 / Liquid 2 (1015 °C), Y_2_O_3_ / Y_2_Cu_2_O_5_ / BaY_2_CuO_5_ / Liquid (1048 °C), Ba_4_YCu_3_O*_x_* / “BaCuO_2_” / Ba_2_YCu_3_O_6+_*_x_*/Liquid (971 °C), BaY_2_CuO_5_ / Ba_2_YCu_3_O_6+_*_x_* / CuO / Liquid (947 °C), BaY_2_CuO_5_ / CuO / Y_2_Cu_2_O_5_ / Liquid (967 °C), Y_2_Cu_2_O_5_ / CuO / Cu_2_O / Liquid, “BaCuO_2_” / Ba_2_YCu_3_O_6+_*_x_* / Liquid 1 / Liquid 2 (973 °C), and Ba_2_YCu_3_O_6+_*_x_* / “BaCuO_2_” / CuO / Liquid (923 °C). With the exception of the first (1274 °C) ternary equilibrium, all occur at a mole fraction of less than 2.0 % 1/2Y_2_O_3_.

The cotectic lines connecting the analytically determined invariant melt compositions have been drawn within the constraints of the experimental uncertainties. They form a self-consistent set in terms of the thermodynamic requirements governing their intersections. The average slopes of the cotectic lines separating the crystallization fields in the lower part of the diagram are defined by the measured melt compositions. In the CuO-rich region of this part of the diagram the location of the phase fields is similar to that of the diagram calculated by Rian [20] using thermodynamic data, especially with regard to the low yttria contents. The cotectic lines in the upper part of the diagram involving BaY_2_O_4_/Y_2_O_3_, Y_2_O_3_/Y_2_Cu_2_O_5_, and Y_2_Cu_2_O_5_/Cu_2_O are fixed only at their lower ends, and therefore their slopes are not yet defined and can only be estimated.

### 3.7 Topological Sequence of Melting Reactions

For clarity, nine of the ten ternary invariant equilibria of Fig. 3 are described in terms of schematic isothermal sections at the appropriate temperatures in Figs. 9a through 9i. The tenth ternary equilibrium, involving Y_2_Cu_2_O_5_/CuO/Cu_2_O/Liquid, was not investigated in our study, but has been estimated, based in part on literature data [2, 54].

In the isothermal sections, a split scale is used as indicated by the break in the side axes. This is necessary in order to give a more comprehensive view of the solids participating in the equilibria, while presenting the schematic details of the participating liquids near the base in expanded form. Reactions discussed are limited to those which are relevant to the construction of the primary phase field of Ba_2_YCu_3_O_6+_*_x_*.

#### 3.7.1 Figure 9a: 923 °C

The initial liquidus event in the system is the eutectic melting of the Ba-Y-Cu-O system. The details of the equilibria associated with the minimum melting have been reported elsewhere [43]. At approximately 923 °C, quenched samples consisting initially of the three phases BaCuO_2_, CuO, and Ba_2_YCu_3_O_6+_*_x_* appeared to have melted slightly, i.e., a small amount of dark stain on the MgO crucible was observed. The x-ray results indicated an additional phase of the reduced BaCu_2_O_2_ type, formed together with CuO, as the melt crystallized on the quench. This reaction represents the eutectic reaction between the three initial phases according to:

$$“{\text{BaCuO}}_{2}”+\text{CuO}+{\text{Ba}}_{2}{\text{YCu}}_{3}O_{6+x}\to L_{1}+O_{2}.$$

This eutectic melting reaction has been observed by numerous phase equilibrium investigators [1, 5, 6, 12, 19]. We have analyzed the liquid participating in this equilibrium and found it to contain a mole fraction of 0.5 % 1/2Y_2_O_3_. The eutectic temperature obtained in this study is higher than typical literature values [5, 12, 19] which was because a fully oxidized “BaCuO_2_” phase was used, and this represents a situation more closely approaching equilibrium [20]. At temperatures above the eutectic, the liquid field expands, mostly in the BaO direction. Based on hydrogen reduction studies, this liquid is reduced relative to the primary solids, with most of the copper in the 1+ valence state. These and related melts are highly fluid and wet the crucible surface extensively, causing the melt to creep out of the crucible.

#### 3.7.2 Figure 9b: 947 °C

The next event to occur with increasing temperature was the disappearance of the tie line between Ba_2_YCu_3_O_6+_*_x_* and CuO, and its replacement by a tie line between BaY_2_CuO_5_ and the newly-formed liquid. A melting event was indicated by DTA to be at around 947 °C, and the x-ray diffraction patterns obtained for a sample equilibrated and quenched at 948 °C revealed the presence of BaY_2_CuO_5_, BaCu_2_O_2_ and CuO. The two latter phases formed as the melt crystallized. This melting reaction is represented according to:

$${\text{Ba}}_{2}{\text{YCu}}_{3}O_{6+x}+\text{CuO}\to {\text{BaY}}_{2}{\text{CuO}}_{5}+L_{1}+O_{2}.$$

This reaction has been reported by other researchers [5, 12, 19] to occur at from 935 °C to 958 °C. The liquid involved has slightly more yttria than the previous reaction, nearly three-quarters of a mol % 1/2Y_2_O_3_. From TGA data, the production of this liquid also involved the loss of oxygen. Schematically, the liquid field is shown as expanding along the BaO-CuO join. Reactions such as this can be described in terms of tie-line switching. The dashed lines in the following isothermal sections are used to indicate the disappearing tie-line for combinations that have become unstable, and the dotted lines represent the thermodynamically more stable pair at higher temperature.

#### 3.7.3 Figure 9c: 967 °C

As the temperature was increased to 967 °C, a melting reaction took place between BaY_2_CuO_5_ and CuO. The x-ray pattern of a sample quenched at 971 °C showed the presence of Y_2_Cu_2_O_5_, which was interpreted as a primary solid phase, together with BaCu_2_O_2_ and CuO crystallized from the melt. Thus the reaction and CuO crystallized from the melt. Thus the reaction observed in the XRD patterns and confirmed by DTA/TGA to occur at 967°C was

$${\text{BaY}}_{2}{\text{CuO}}_{5}+\text{CuO}\to Y_{2}{\text{Cu}}_{2}O_{5}+L_{1}+O_{2}.$$

This reaction has also been observed in other investigations [5, 12, 19], where it has been reported to occur at 972 °C – 975 °C. The liquid was analyzed and found to contain a mole fraction of 0.6 % 1/2Y_2_O_3_. In Fig. 9c the overall liquid region has expanded considerably and encompasses a range of BaO/CuO compositions, yet the yttria content is still low. At this temperature a sizeable range of liquid compositions coexists with the Ba_2_YCu_3_O_6+_*_x_* phase, again, all with low yttria content.

#### 3.7.4 Figure 9d: 971 °C

At 971°C, the first event involving Ba_2_YCu_3_O_6+_*_x_* in the left hand side of the diagram occurred. This reaction was determined using BaO-derived starting materials and includes Ba_4_YCu_3_O*_x_*, Ba_2_YCu_3_O_6+_*_x_* “BaCuO_2_” and L_2_, according to

$${\text{Ba}}_{4}{\text{YCu}}_{3}O_{x}+“{\text{BaCuO}}_{2}”\to {\text{Ba}}_{2}{\text{YCu}}_{3}O_{6+x}+L_{2}+O_{2}.$$

This liquid was the most Ba-rich of any on the diagram, with a Ba:Y:Cu amount of substance ratio of 55.5: 1.6: 42.9. This liquid lies in the Ba_2_CuO_3_–“BaCuO_2_” –Ba_4_YCu_3_O*_x_* compositional region, although the bulk composition was in the Ba_4_YCu_3_O*_x_* – Ba_2_YCu_3_O_6+_*_x_* – “BaCuO_2_” phase triangle. A reaction involving the same three solids plus liquid was reported by Osamura and Zhang [12] and Krabbes et al. [19] to occur at 975 °C, however their reaction was instead written with Ba_2_YCu_3_O_6+_*_x_* plus BaCuO_2_ on the left side and Ba_4_YCu_3_O*_x_* plus Liquid on the right.

When our starting materials were prepared from carbonates and annealed in pure air, the initial event in this part of the system occurred at a much higher temperature (1005 °C), which was near the melting point of Ba_2_YCu_3_O_6+_*_x_*. The reaction involved Ba_4_YCu_3_O*_x_* (possibly an oxycarbonate, as discussed by Roth et al. [1]), “BaCuO_2_,” BaY_2_CuO_5_ and L_2_, but not Ba_2_YCu_3_O_6+_*_x_*, as shown in the inset of Fig. 3. The implication is that the presence of carbonate somehow stabilizes the “BaCuO_2_” + BaY_2_CuO_5_ pair at the expense of the Ba_2_YCu_3_O_6+_*_x_* + Ba_4_YCu_3_O*_x_* pair. This suggests that “BaCuO_2_” and/or BaY_2_CuO_5_ are capable of incorporating more carbonate than the alternative pair, possibly also as an oxycarbonate solid solution.

#### 3.7.5 Figure 9e: 972 °C

At a slightly higher temperature, again using BaO-derived starting materials, the tie line “BaCuO_2_” + Ba_2_YCu_3_O_6+_*_x_* gives way to L_1_ + L_2_ according to the reaction:

$$“{\text{BaCuO}}_{2}”+{\text{Ba}}_{2}{\text{YCu}}_{3}O_{6+x}\to L_{1}+L_{2}+O_{2}.$$

This reaction marks the first coexistence of L_1_ and L_2_, and therefore is the onset of liquid immiscibility in the system. With increasing temperature, the two liquid field spreads in both directions, toward the BaO-CuO edge, which is nearby, and in the other direction towards Ba_2_YCu_3_O_6+_*_x_*, leading to the melting of Ba_2_YCu_3_O_6+_*_x_*. As noted above, a composition corresponding to the L_2_ rich side of the pair has been measured, and has a mole fraction 1/2Y_2_O_3_ of <0.2 %. This places it very close to the appearance of the two liquid field on the BaO-CuO join, in reasonable agreement with data of Zhang et al. [53].

#### 3.7.6 Figure 9f: 973 °C

At a slightly higher temperature (in practice the temperatures of Figs. 9d, 9e and 9f were difficult to distinguish) and using BaO-derived starting materials, a reaction between Ba_4_YCu_3_O*_x_*, Ba_2_YCu_3_O_6+_*_x_*, BaY_2_CuO_5_ and L_2_ occurred as follows:

$${\text{Ba}}_{4}{\text{YCu}}_{3}O_{x}+{\text{Ba}}_{2}{\text{YCu}}_{3}O_{6+x}\to {\text{BaY}}_{2}{\text{CuO}}_{5}+L_{2}+O_{2.}$$

This reaction must take place before the melting of Ba_2_YCu_3_O_6+_*_x_* to BaY_2_CuO_5_ plus two liquids can occur. Due to the small amount of liquid produced by the reaction and difficulties in sample preparation, no direct compositional measurement of this melt was made, but its position on the diagram is shown so as to be topologically consistent with the other equilibria. A reaction involving the same three solids plus liquid was reported by Osamura and Zhang [12] at 1010 °C, but their reaction was written as the incongruent melting of Ba_2_YCu_3_O_6+_*_x_* to Ba_4_YCu_3_O*_x_* plus BaY_2_CuO_5_ plus Liquid. We favor a different melting reaction for Ba_2_YCu_3_O_6+_*_x_*, as already discussed.

#### 3.7.7 Figure 9g: 1015 °C

The high *T*_c_ phase Ba_2_YCu_3_O_6+_*_x_* melts over a small range of approximately 1010 °C – 1015 °C according to

$${\text{Ba}}_{2}{\text{YCu}}_{3}O_{6+x}\to {\text{BaY}}_{2}{\text{CuO}}_{5}+L_{1}+L_{2}+O_{2}.$$

The melting of Ba_2_YCu_3_O_6+_*_x_* is completed at 1015 °C. As mentioned earlier, analyzed liquid compositions quenched just above this melting point do not lie on the extension of the tie line between Ba_2_YCu_3_O_6+_*_x_* and BaY_2_CuO_5_, but fall off this join to the copper rich side; they contain a mole fraction of less than 1 % 1/2Y_2_O_3_ (Table 2). The melting of a ternary solid to three phases is a general feature of ternary incongruency, and there is no requirement for the liquid to lie on this extension. The occurence of L_2_ in the melting reaction of Ba_2_YCu_3_O_6+_*_x_* has been difficult to detect because of the smaller amount produced relative to L_1_ and also because the latter may preferentially penetrate the wick. However, a liquid on the BaO-rich side of the gap has indeed been observed by itself as a participant in the equilibrium at 971 °C described above. Other researchers [24, 25] using a “dip” sampling method may have observed a melt composition on the BaY_2_CuO_5_ – Ba_2_YCu_3_O_6+_*_x_* extension because their method sampled both liquids simultaneously, under conditions which did not allow the liquids to segregate.

#### 3.7.8 Figure 9h: 1048 °C

When the temperature is raised still further, the two liquid region expands to the BaO-CuO join, and then is bridged over. At 1048 °C a tie line switch from BaY_2_CuO_5_ plus Y_2_Cu_2_O_5_ to Y_2_O_3_ plus liquid takes place:

$${\text{BaY}}_{2}{\text{CuO}}_{5}+Y_{2}{\text{Cu}}_{2}O_{5}\to Y_{2}O_{3}+L+O_{2}.$$

The x-ray analysis results indicated the presence of Y_2_O_3_, and BaCu_2_O_2_ which was crystallized from the melt. The melt composition showed a substantial increase in the content of BaO, relative to the 967 °C reaction, so that the liquid has moved toward the barium rich side of the diagram, and this reaction involves a liquid with a mole fraction of about 1.25 % 1/2Y_2_O_3_. This reaction was reported by Osamura and Zhang [12] and Krabbes et al. [19] to occur at 1027 °C and 1061 °C, respectively.

#### 3.7.9 Figure 9i: 1274 °C

A DTA study of the melting of BaY_2_CuO_5_, the “green phase,” indicated the melting event to occur at an initial temperature of about 1270 °C, with completion at about 1274 °C. As described above, BaY_2_CuO_5_ was found to melt according to the following reaction:

$${\text{BaY}}_{2}{\text{CuO}}_{5}\to {\text{BaY}}_{2}O_{4}+Y_{2}O_{3}+L+O_{2}.$$

It was assumed in constructing Figure 9i that the two liquid field has been bridged over at a temperature below 1274°C. Similarly, the extent of the liquid along the CuO-Y_2_O_3_ join is estimated. The Y_2_O_3_ field at this temperature apparently includes most of the liquidus on the CuO-rich side of the diagram, as there are no other solids which have not melted in this region.

### 3.8 Verification of the Ba_2_YCu_3_O_6+_*_x_* Phase Field

In order to confirm the approximate lateral extent of the Ba_2_YCu_3_O_6+_*_x_* primary phase field as shown in Fig. 3, two compositions, labelled “B” (amount of substance ratio Ba:Y:Cu = 51.00 : 1.50 : 47.50), and “C” (amount of substance ratio Ba:Y:Cu = 26.85 : 0.65 : 72.50) in Fig. 7 were prepared using BaO-derived starting materials. Composition B plots in the Liquid 1 part of the Ba_2_YCu_3_O_6+_*_x_* field, and composition C plots in the Liquid 2 region. Crystallization experiments in the DTA/TGA apparatus in purified air were completed on these compositions as follows. Carefully homogenized samples were annealed at 850 °C, and heated to 1050 °C until weight had stabilized; at this point the samples had completely melted. Composition B was subsequently cooled to 1015 °C, and from that point slowly cooled at 0.2 °C /min to 935 °C, then the furnace power was shut off. Composition C was cooled from 1050 °C to 1025 °C and slowly cooled at 0.2 °C/min to 985 °C, followed by power shutoff. Both experiments were designed so that a temperature drop of only 10 °C to 15 °C was required to solidify the remaining melt. This was accomplished in less than a minute once the power was shut off.

**Fig. 10:**
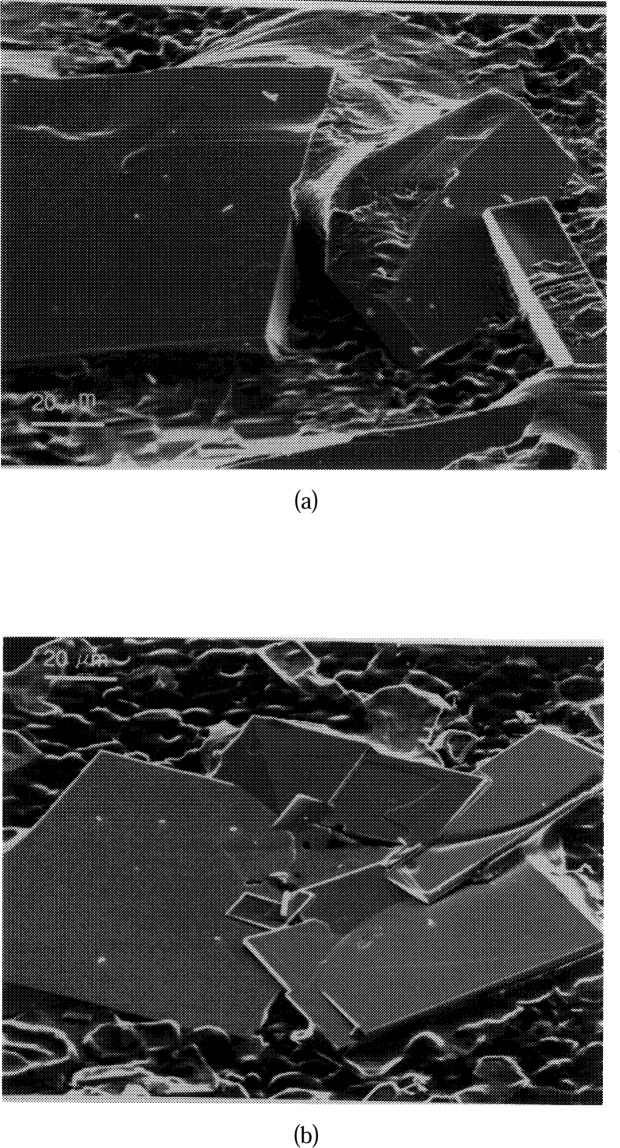
Secondary electron images of Ba_2_YCu_3_O_6+_*_x_* crystallization experiments in (a) the L_1_ region (composition B, Fig. 7), and (b) the L_2_ region (composition C, Fig. 7).

Results are shown in Figs. 10a and 10b, where it can be seen that flat crystals of Ba_2_YCu_3_O_6+_*_x_* (determined by EDS) 100 μm to 200 μm across were formed in both experiments. Because of their large size and well-developed morphology, these crystals could be readily differentiated from the surrounding melt, and are interpreted as having crystallized in equilibrium with the melt. Similar crystals of other phases were not observed. At equilibrium, within the primary Ba_2_YCu_3_O_6+_*_x_* phase field, it must be true that Ba_2_YCu_3_O_6+_*_x_*, and only Ba_2_YCu_3_O_6+_*_x_*, crystalllizes from the melt. The primary phase field is bounded by cotectic lines along which Ba_2_YCu_3_O_6+_*_x_* crystallizes in equilibrium with CuO, “BaCuO_2_,” BaY_2_CuO_5_, or Ba_4_YCu_3_O*_x_*. Outside the primary phase field, at equilibrium, Ba_2_YCu_3_O_6+_*_x_* cannot crystalllize directly from the liquid by itself. Since we have observed only Ba_2_YCu_3_O_6+_*_x_* crystals forming from the melts of compositions B and C, we conclude that compositions B and C lie within the primary Ba_2_YCu_3_O_6+_*_x_* phase field. This provides a first order verification of the Ba_2_YCu_3_O_6+_*_x_* primary field in Fig. 3.

Certain of the Ba_2_YCu_3_O_6+_*_x_* crystals produced in the composition B experiment had poorly defined edges, sometimes with a reentrant configuration relative to the surrounding melt. This is believed to represent resorption of Ba_2_YCu_3_O_6+_*_x_* during the final segment of the crystallization path, as the “BaCuO_2_”–Ba_2_YCu_3_O_6+_*_x_* cotectic was intersected (Fig. 7). By contrast, the composition C crystals showed only straight boundaries, and rather than resorption, an abundance of sharply stepped terraces suggested the crystals were undergoing active growth at the moment the experiment was terminated. It is concluded that the crystallization path in the composition C experiment did not reach the “BaCuO_2_”-Ba_2_YCu_3_O_6+_*_x_* cotectic boundary (Fig. 7).

### 3.9 Oxidation/Reduction Nature of Melt

For samples with melts in Fig. 3, substantial weight loss was observed associated with the DTA/TGA events on the heating cycles. This indicated oxygen loss, and that the oxidation-reduction process played an important role in the melting reactions in this region of the Ba-Y-Cu-O system.

Data on the copper oxidation state of melts are sum-marized in Table 3. Hydrogen reduction was completed on the melt of the BaY_2_CuO_5_ phase, which had the highest melting temperature of the reaction sequence (1274 °C). In this reaction, the starting material contained copper as almost 100 % Cu^+2^, while in the melt, the copper was almost totally reduced to Cu^+1^, with an amount of substance ratio *n*(Cu^+1^)/*n*(Cu^+1^ + Cu^+2^) of 0.99. Compositions B and C (Fig. 7) were melted at a much lower temperature, 1050 °C, and consequently the oxygen loss associated with melting was not as severe. For these melts, hydrogen reduction was not necessary, as the oxidation state of the starting materials was well known (essentially all Cu^+2^), and the weight loss associated with complete melting could be determined thermogravimetrically. Melt B (Liquid 1) showed a *n*(Cu^+1)^/*n*(Cu^+1^ + Cu^+2^) amount of substance ratio of 0.57, whereas melt C (Liquid 2) had a *n*(Cu^+1^)/*n*(Cu^+1^ + Cu^+2^) amount of substance ratio of 0.39. Liquid 2 was therefore substantially more oxidized than Liquid 1, which may be an important factor in the existence of the miscibility gap.

### 3.10 Applications

The quantitative melt data reported in this paper are important for crystal growth and for melt processing. Because of the relatively narrow (in terms of the Y_2_O_3_ component) primary phase field of Ba_2_YCu_3_O_6+_*_x_*, the stoichiometry must be precisely controlled to maximize the yield by the single crystal growth technique. Furthermore, these melts have low yttrium content, indicating the growth rate and the size of single crystals may be restricted as a result.

The observation of immiscible liquids should be considered in designing processing paths, such as crystal growth, melt processing, and solidification. It appears that two different liquid regions, instead of just one, are available for the crystallization of Ba_2_YCu_3_O_6+_*_x_*. It is important to know how these two liquids differ in their properties and which is best for crystal growth. The present paper provides the basic information for crystallization alternatives. From the compositional point of view, the second liquid (L_2_) has a slightly higher yttrium content, and thus could in principle offer increased growth rates, other properties being equal. Another advantage of the second liquid may be the possibility of a longer crystallization path and therefore increased yield, in that more Y is extracted to form Ba_2_YCu_3_O_6+_*_x_*. This is a consequence of the fact that the L_2_/Ba_2_YCu_3_O_6+_*_x_* field is broader in the direction of the Ba_2_YCu_3_O_6+_*_x_* crystallization vector and that it extends almost completely to the BaO-CuO edge, thus allowing for nearly complete Y depletion (Fig. 7).

Because the loss of oxygen accompanies most melting, this indicates that during the crystallization process, the melt needs to regain adequate oxygen. As a result, *p*(O_2_) can be used as an additional control variable during melt processing, provided that a means of overcoming the kinetic barrier to reoxidation can be devised. Further work is needed to study the effect of oxygen partial pressure on the primary phase field of Ba_2_YCu_3_O_6+_*_x_*. It is possible that the Y content may be higher at higher oxygen partial pressures, or the area of the primary phase field may be expanded towards the higher Y side at higher oxygen content. In particular, the effect of O_2_ pressure on the field of liquid immiscibility must be investigated.

## 4. Summary

Experimental apparatus and procedures for the determination of the oxygen and cation stoichiometry of liquids participating in the melting reactions of the Ba-Y-Cu-O system have been developed and applied. The topological sequence of melting in purified air, as a function of temperature, has been described, from the minimum melting of the system through melting of the “green phase,” BaY_2_CuO_5_. The melting sequence as a function of temperature is more complicated than those reported in literature. This sequence differs from the previously published ones in several specific respects which can be summarized as follows: (1) it is suggested that BaY_2_CuO_5_ (“green phase”) melts to BaY_2_O_4_+Y_2_O_3_+Liquid instead of to Y_2_O_3_+Liquid; (2) it is postulated that Ba_2_YCu_3_O*_x_* melts to BaY_2_CuO_5_+Liquid_1_+Liquid_2_, instead of to BaY_2_CuO_5_ +Liquid; (3) evidence shows that at temperatures near its melting point, BaCuO_2_ is nonstoichiometric in Ba and Cu.

The quantitative melt analyses indicate the primary phase fields are relatively large for the nonsuperconductors, especially Y_2_O_3_. The large areas of these phase fields are in contrast with the narrow extent in Y_2_O_3_ content of the primary field of the high *T*_c_ Ba_2_YCu_3_O*_x_* phase. The latter has a width of only a mole fraction of about 0.3 % 1/2Y_2_O_3_ at the CuO-rich end, which broadens to a mole fraction of about 2.0 % 1/2Y_2_O_3_ at the BaO-rich limit. This thin slice extends from a mole fraction of 43 % CuO to to a mole fraction of 76 % CuO, and is divided roughly in half by a postulated liquid miscibility gap. In general, our current diagram differs from most other experimental diagrams published in terms of the location, size, and shape of primary fields of various phases; however, at the CuO-rich end, it agrees relatively well with a version calculated from thermodynamic data [20].

The observation of the presence of two liquids may have significant impact on the areas of melt processing and crystal growth. This important phenonmenon still needs further understanding and characterization. Further work is also needed to study the primary phase field of the Ba_2_YCu_3_O_6+_*_x_* phase in terms of different oxygen partial pressures.

## Figures and Tables

**Fig. 9a:**
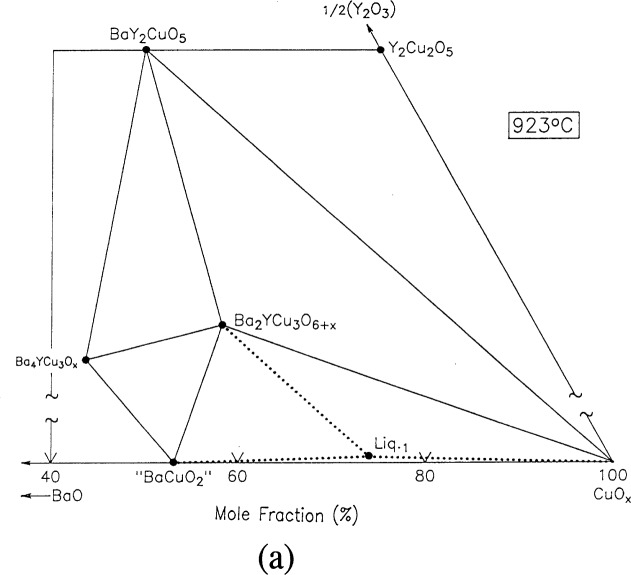
Ba-Y-Cu-O melting reacion in the vicinity of the Ba_2_YCu_3_O_6+_*_x_* primary field at 923 °C.

**Fig. 9b:**
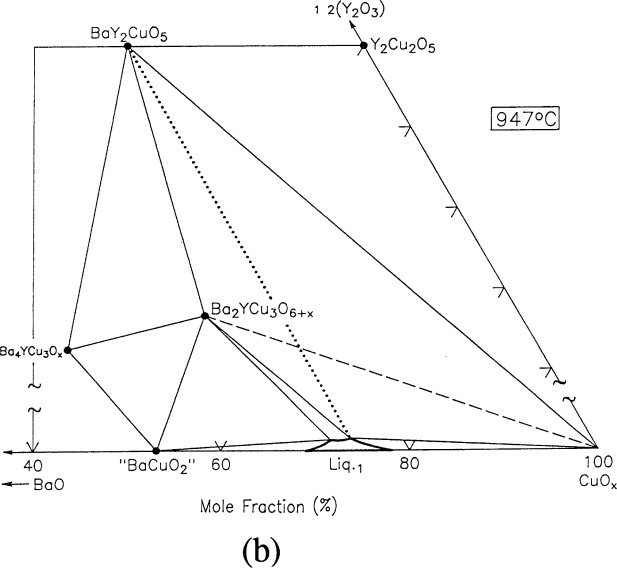
Same as Fig. 9a at 947 °C.

**Fig. 9c:**
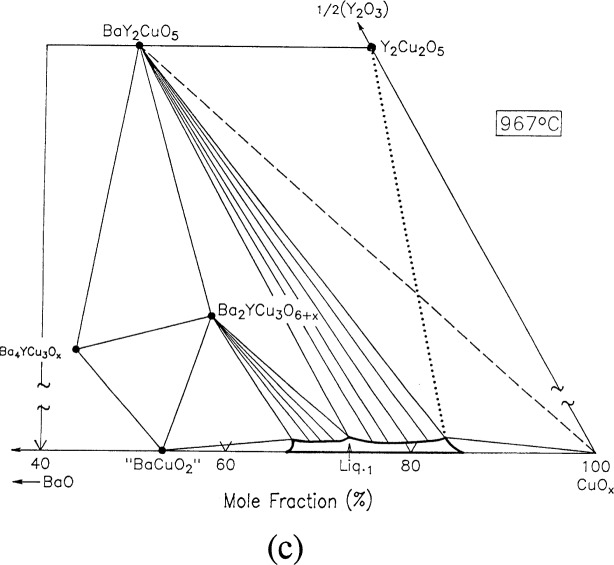
Same as Fig. 9a at 967 °C.

**Fig. 9d:**
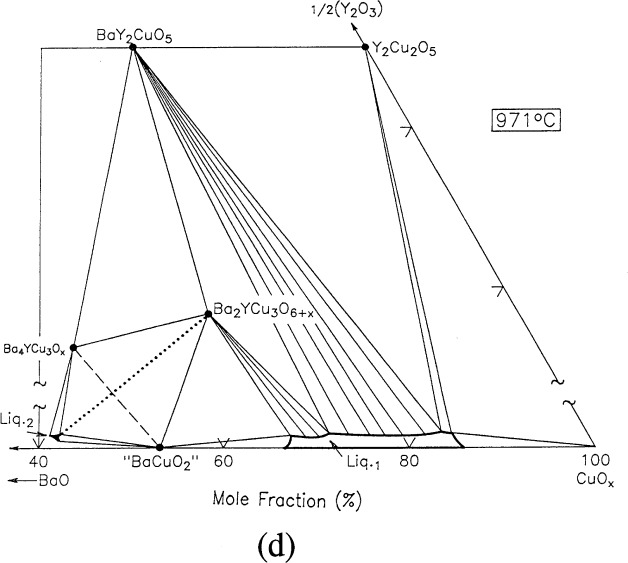
Same as Fig. 9a at 971 °C.

**Fig. 9e:**
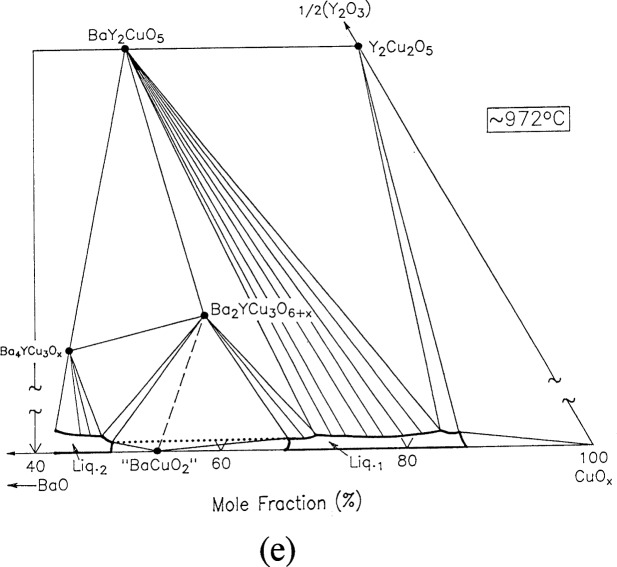
Same as Fig. 9a at 972 °C.

**Fig. 9f:**
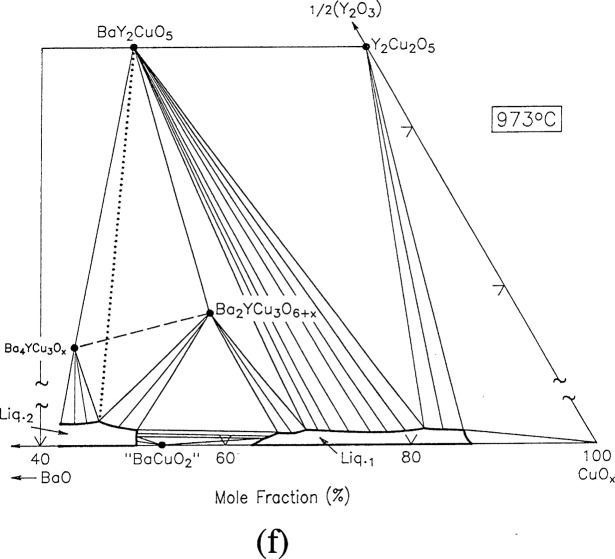
Same as Fig. 9a at 973 °C.

**Fig. 9g:**
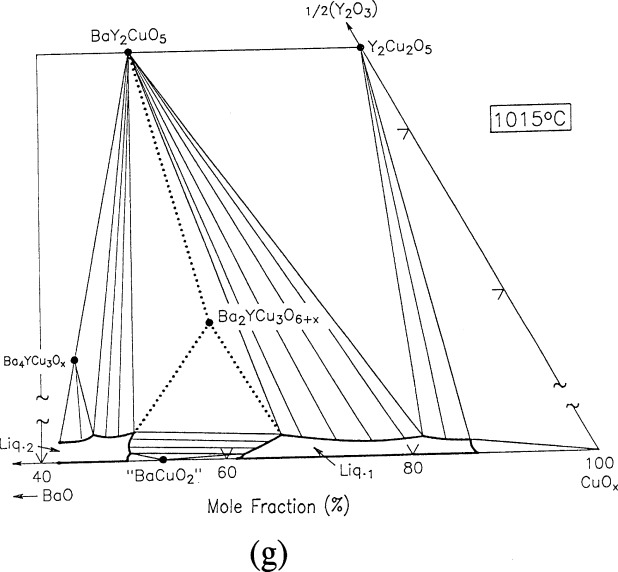
Same as Fig. 9a at 1015 °C.

**Fig. 9h:**
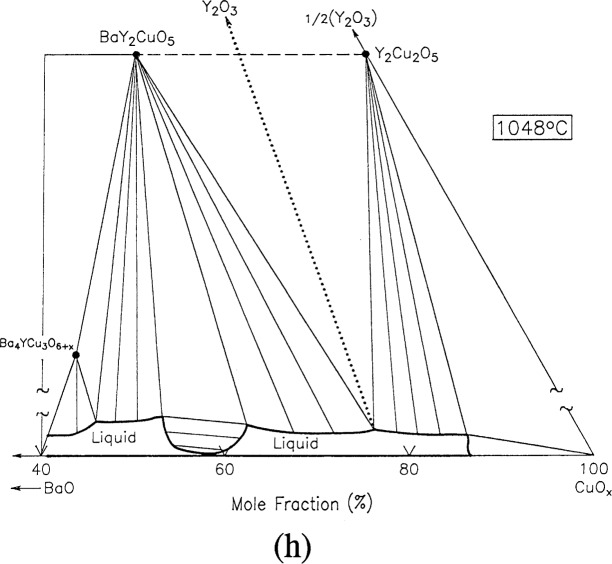
Same as Fig. 9a at 1274 °C.

**Fig. 9i:**
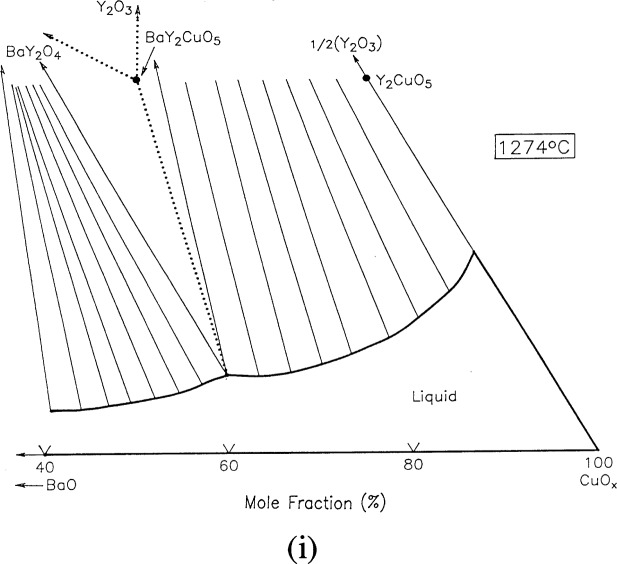
Same as Fig. 9a at 1274 °C.

**Table 1:** Relevant compositions (mole fraction, *x*_B_) prepared and their initial melting temperatures. “BaCuO_2_” refers to Ba_0.94_Cu_1.06_O*_x_*. (The expanded uncertainties in sample compositions are ±0.01 *x*_B_)

Sample No.	*x*_B_			DTA temperature (°C)	Subsolidus phase field
	BaO (%)	1/2Y_2_O_3_ (%)	CuO (%)		
1	23.0	4.0	73.0	923	Ba_2_YCu_3_O_6+_*_x_* – BaCuO_2_ – CuO
2	35.0	6.0	59.0	926	Ba_2_YCu_3_O_6+_*_x_* – BaCuO_2_ – CuO
3	23.0	5.0	72.0	923	Ba_2_YCu_3_O_6+_*_x_* – BaCuO_2_ – CuO
4	33.33	0.0	66.67	931	BaCuO_2_ – CuO
5	25.0	12.5	62.5	947	Ba_2_YCu_3_O_6+_*_x_* – CuO
6	12.5	25.0	62.5	967	BaY_2_CuO_5_ – CuO
7^a^	45.45	9.1	45.45	971	Ba_2_YCu_3_O_6+_*_x_* – Ba_4_YCu_3_O*_x_* – “BaCuO_2_”
8^a^	45.9	1.3	52.8	972	Ba_2_YCu_3_O_6+_*_x_* – “BaCuO_2_”
9^a^	47.00	0.0	53.00	984	“BaCuO_2_”
10	41.7	6.5	51.8	1005	Ba_2_YCu_3_O_6+_*_x_* – “BaCuO_2_”
11	50.0	0.0	50.0	1010	BaCuO_2_
12	33.33	16.67	50.0	1010	Ba_2_YCu_3_O_6+_*_x_*
13	37.5	25.0	37.5	1023	BaY_2_CuO_5_ – BaCuO_2_
14	12.5	50.0	37.5	1048	BaY_2_CuO_5_ – Y_2_Cu_2_O_5_
15	41.7	25.0	33.3	1070	Ba_4_YCu_3_O*_x_* + BaY_2_CuO_5_
16	50.0	12.5	37.5	1080	Ba_4_YCu_3_O*_x_*
17	25.0	50.0	25.0	1270	BaY_2_CuO_5_

a Indicates starting materials prepared using BaO; all others used BaCO_3_

**Table 2:** Compositions of liquids (mole fraction, *x*_B_) produced by invariant (in air) melting reactions, and phases present in quenched residual

Sample No.	Anneal/Quench temperature (°C)	*x*_B_			Phases present in residual^a^
		Liquid composition			
		BaO (%)	CuO (%)	1/2Y_2_O_3_ (%)	
1	935	23.2	76.3	0.5	Ba_2_YCu_3_O*_x_*, BaCuO_2_, CuO, BaCu_2_O_2_
4	936	16.3	83.7	–	BaCuO_2_, CuO, BaCu_2_O_2_
5	948	5.1	74.2	0.7	BaY_2_CuO_5_, BaCu_2_O_2_, CuO
6	971	13.9	85.5	0.6	Y_2_Cu_2_O_5_, BaCu_2_O_2_, CuO
7	976^b^	55.7	42.9	1.4	Ba_2_YCu_3_O*_x_*, BaCuO_2_, Ba_4_YCu_3_O*_x_*
8	978^b^	49.7	50.1	<0.2	Ba_2_YCu_3_O*_x_*, BaCuO_2_, Ba_4_YCu_3_O*_x_*
10	1005	55.1	44.6	0.3	BaY_2_CuO_5_, BaCuO_2_, BaCu_2_O_2_
12	1011	33.3	66.0	0.7	BaY_2_CuO_5_, BaCuO_2_, BaCu_2_O_2_
14	1051	21.6	77.2	1.2	Y_2_O_3_, BaCu_2_O_2_, Cu_2_O
17	1274	37.9	59.0	3.1	Y_2_O_3_, BaY_2_O_4_, BaCu_2_O_2_

a Determined by x-ray analysis. These analyses include phases stable at the temperature of the experiment plus melt crystallization products.

b Samples No.7 and No. 8 were furnace cooled; all others were quenched.

**Table 3:** Oxidation states of liquids, as calculated from TGA data

Reaction or compositional point	Temperature °C	*x*_B_			*n* (Cu^+1^)/*n* (Cu^+1^ + Cu^+2^)
		Composition (mole fraction, *x*_B_)			
		BaO (%)	1/2 Y_2_O_3_ (%)	CuO (%)	
BaY_2_CuO_5_/Y_2_O_3_/BaY_2_O_4_/Liquid	1274 °C	37.9	3.1	59.0	0.99
B (see Fig. 7)	1050 °C	26.85	0.65	72.50	0.57
C (see Fig. 7)	1050 °C	51.00	1.50	47.50	0.39
